# Catheter-Directed Thrombolysis in the treatment of acute Portomesenteric Vein Thrombosis after Laparoscopic Sleeve Gastrectomy

**DOI:** 10.1186/s12959-022-00415-w

**Published:** 2022-09-29

**Authors:** Ali Barah, Israa Al-Hashimi, Rahil Kassamali, Qayed Aldebyani, Omran Almokdad, Ayman Elmagdoub, Mohammed Khader, Saad U. Rehman, Ahmed Omar

**Affiliations:** Clinical Imaging Department, Hamad Medical Corporation, Doha, Qatar

**Keywords:** Laparoscopic Sleeve Gastrectomy, Portomesenteric Vein Thrombosis, Catheter-Directed Thrombolysis

## Abstract

**Background:**

Portomesenteric Vein Thrombosis (PMVT) following Laparoscopic Sleeve Gastrectomy (LSG) is an uncommon but potentially debilitating complication. Catheter-Directed Thrombolysis (CDT) has an evolving role in recanalizing the venous flow and preventing thrombus propagation. Therefore, it can be used as an alternative or in combination with systemic anticoagulants in selected patients. We report two trans-hepatic and trans-splenic CDT. The patient’s clinical details, radiological findings, safety, and efficacy are reported.

**Cases presentation:**

Two patients presented to the Emergency Department (ED) within 14 days of surgery. The presenting complaints were generally nonspecific. The diagnosis of PMVT was established in both patients based on abdominal Contrast-Enhanced Computed Tomography (CECT). The two patients received a combined therapy of subcutaneous (SC) heparinization and CDT using a trans-hepatic approach in case 1 and a trans-splenic approach in case 2. Subsequent post-procedure venograms and CECT were performed and showed significant thrombus resolution. Both patients received oral anticoagulant therapy upon discharge with a successful overall recovery.

**Conclusion:**

PMVT is an infrequent and severe post LSG complication. Various approaches for re-establishing the portal venous flow have been described according to the severity of venous thrombosis. This article describes CDT therapy as a safe and effective option for treating PMVT in symptomatic patients.

## Background

Worldwide, Laparoscopic Sleeve Gastrectomy (LSG) is currently a recognized dominant bariatric procedure [1]. Portomesenteric Vein Thrombosis (PMVT) became a well-recognized yet rare clinical complication of LSG. According to one study, the incidence of PMVT in LSG appears to be higher than the rates of other bariatric operations, with a total population incidence of 1.1% [2]. The late detection and revascularization of PMVT, especially in patients not receiving adequate anticoagulation; can result in devastating complications leading to ascites in 62%, esophageal varices in 58%, terminal gastroesophageal bleeding in 47%, intestinal perforation, infarction, and secondary peritonitis [2]. In addition, long-term sequelae of portal hypertension are found in 50% of patients with PMVT [3]. Early diagnosis and treatment can prevent acute and late complications secondary to PMVT [4, 5]. There is growing attention for endovascular therapy as a practical option in treating patients with extensive PMVT in whom conservative treatment with solely anticoagulation results in inadequate revascularization [5]. Early intervention with combined therapy resolves thrombus, thus improving the symptoms and decreasing the secondary complications of bowel ischemia, portal hypertension, and other serious complications such as death [5, 6]. CDT is considered an effective yet invasive intervention. It requires highly specialized units where interventional radiology specialists can carry it in secondary and tertiary facilities. It is, thus, reserved for acute and subacute cases, including our two patients where early signs of bowel ischemia were observed, increasing the need for more invasive revascularization in the form of combined therapy. The monotherapy treatment was clinically decided to be inadequate to improve the PMVT and its secondary complications [5]. CDT can result in hemorrhagic complications as blood vessels can be damaged during the procedure, as seen in 60% of patients with a mortality rate of up to 21% of patients [7–9]. The mechanical interventions in the CDT, including mechanical thrombectomy can also lead to pulmonary embolism [9].

The purpose of this study was to present cases that developed PMVT after LSG and precisely discuss the effectiveness of CDT in the treatment of acute and subacute cases.

## Case presentation

### Patient # 1

**Fig. 1:**
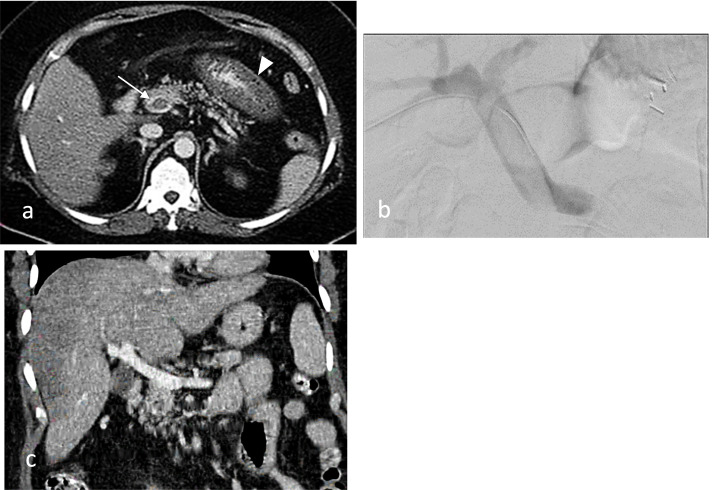
**a** Pre-procedural Contrast-Enhanced Computed Tomography (CECT) showing portal vein thrombosis (arrow) and jejunal wall thickening (arrow-head). **b** Pre-procedural trans-hepatic portogram showing main portal vein thrombosis with patency of intrahepatic portal branches. **c** Follow-up Contrast-Enhanced Computed Tomography (CECT) demonstrating recanalization of the portal vein

A 48-year-old female patient, unknown to have any previous remarkable medical history, presented to our hospital two weeks after LSG, indicated for morbid obesity, with abdominal pain without tenderness or vomiting. CECT revealed complete thrombosis of the superior mesenteric vein (SMV) extending to the main portal vein, thickened proximal jejunal loops with moderate enhancement, and mild adjacent mesenteric stranding. However, the visceral arteries were patent (Fig. 1a). The patient was found to have heterozygous Factor V Leiden mutation, which may have additive effect on the surgical-related provoking factors of PMVT. A combined treatment of SC heparinization by Low Molecular Weight Heparin (LMWH) with local CDT was started. A trans-hepatic approach was performed under ultrasound guidance using an 18-gauge trocar needle. A 5F vascular sheath was inserted over a 0.035-inch hydrophilic guidewire.

The occlusion was then traversed using a 5F angiographic catheter with the same guidewire until reaching the SMV. The angiographic catheter was then replaced by a 5F multi-side-hole infusion catheter. The venogram showed SMV occlusion extended to the main portal vein (Fig. 1b). A loading dose of 5 mg of tissue Plasminogen Activator (tPA) was given through the catheter, followed by continuous infusion of tPA with a rate of 0.5 mg per hour for six hours. The SMV venogram showed partial recanalization of SMV and the main portal vein six hours later. Based on venogram findings and on post-procedural abdominal CECT images, it was decided to continue the tPA infusion at the same rate for 72 h. The follow-up venogram showed significant recanalization of the PMVT. The tPA infusion was then discontinued, and the catheter with the vascular sheath was removed after hepatic tract embolization using an absorbable gelatin sponge. The patient was found to be clinically stable, thus was discharged with oral anticoagulant for three months. A Follow-up abdominal CECT performed ten days in post treatment demonstrated further improvement; however, residual filling defects were still noted in the SMV and main portal vein (Fig. 1c). A three months follow-up with an abdominal ultrasound showed a complete resolution of PMVT.

### Patient # 2

**Fig. 2:**
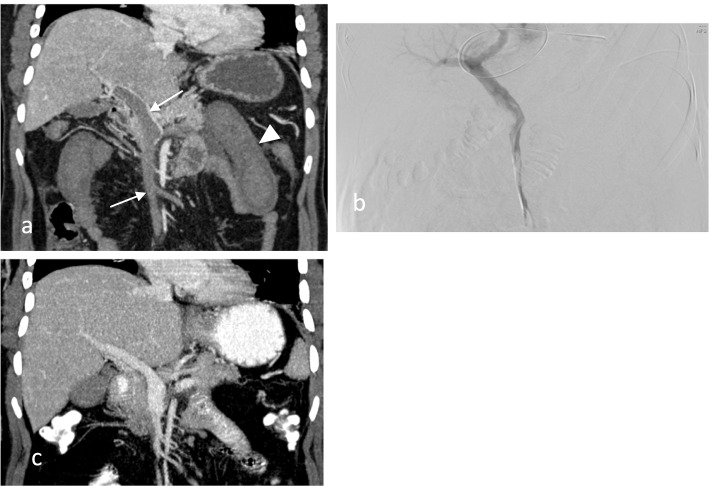
**a** Pre-procedural Contrast-Enhanced Computed Tomography (CECT) showing Portomesenteric Vein Thrombosis (PMVT) (arrows) with small bowel wall thickening (arrow-head). **b** Follow-up trans-splenic portogram demonstrating recanalization of the portal vein with persistence partial thrombosis of superior mesenteric vein (SMV). **c** One week post-procedural Contrast-Enhanced Computed Tomography (CECT) showing recanalization of the portal vein and mesenteric vein (SMV)

A 35-year-old male patient with ten-day history of LSG, unknown to have a previous remarkable medical history including thromboembolic events, presented in ED with diffuse abdominal pain and mild abdominal tenderness. Laboratory results demonstrated a raised white blood cell count. Abdominal ultrasound was performed, and it showed no evidence of flow in the portal vein, suggesting portal vein thrombosis. An abdominal CECT was performed, showing evidence of persistent SMV thrombosis extending to the intrahepatic portal veins with remarkable small bowel thickening (Fig. 2a). A systemic SC heparinization by LMWH combined with CDT was decided to be commenced. Due to the total occlusion of intrahepatic portal veins, a trans-splenic approach was selected. Under ultrasound guidance, percutaneous trans-splenic access to the main splenic vein was obtained by puncturing a perihilar splenic vein with a 21-gauge Chiba needle (Cook; Bloomington; IN). A 5F vascular sheath was then inserted over a 0.018-inch guidewire. Next, a 5F angiographic catheter over a 0.035-inch hydrophilic guidewire was used to cross the thrombus until reaching the SMV. The angiographic catheter was then exchanged with 5F multi-side hole perfusion catheter with the tip placed in the SMV. An initial dose of 5 mg of tPA was started, followed by continuous tPA infusion with a rate of 0.5 mg per hour. The tPA infusion was continued for more than 24 h, and the follow-up venogram demonstrated partial recanalization of SMV and portal veins (Fig. 2b). The tPA infusion was then discontinued, and the splenic tract was embolized using absorbable gelatin sponge while pulling back the vascular sheath. The patient underwent limited bowel resection secondary to the mesenteric vein infarction. One week later, an abdominal CECT follow-up was performed and revealed further patency of the partial thrombus involving intrahepatic branches of the main portal vein and SMV with no ongoing evidence of bowel ischemia (Fig. 2c). The patient was discharged after a good recovery with oral anticoagulant therapy of three months duration.

## Discussion and conclusions

PMVT has become a potentially severe reported complication of LSG despite its rare frequency [6, 10]. In our case series, one patient was found to have heterozygous Factor V Leiden mutation suggesting an additive cause of PMVT. However, in post LSG, the etiology of PMVT is multifactorial and can have additional intraoperative and post-operative factors [11, 12].

LSG and other laparoscopic bariatric procedures can have intraoperative factors using mechanical and thermal energy devices, potentially contributing to thrombus formation. Also, the liver’s prolonged retraction can result in venous stasis resulting in clot formation [12]. In addition, the CO2 insufflation during the initiation of a laparoscopic procedure can increase the intra-abdominal pressure, which decreases the splanchnic and portal vein blood flow and results in thrombotic events in the territories [12]. Finally, post-operative factors usually present after the patient discharge and include dehydration and inadequate thromboprophylaxis measures [4, 12].

CECT is considered a gold standard in detecting PMVT and bowel ischemia, with a sensitivity reaching 90% [13]. In our cases, CECT showed high accuracy in detecting the extent of PMVT and the degree of bowel insult, which determined the type of management and allowed us to plan the access to the portal system.

The management of PMVT depends on many factors such as the extension of thrombus, presence or not of bowel ischemia, and patient’s general condition. The primary goal is to recanalize the affected veins to prevent secondary complications and further thrombus extension.

There is remarkable poverty in the studies comparing the various recanalization modes used in PMVT. This can be achieved through systemic anticoagulation, CDT, combination therapy, or surgical interventions [14]. Systemic anticoagulation should be immediately initiated in all cases. It can solely achieve complete recanalization, especially in the nonocclusive non-ischemic bowel.

In up to 80% of cases, it was found that long-term anticoagulation alone for a minimum of six months may also recanalize partial to complete thrombosis [15]. While in another study, it was indicated that approximately 30 to 50% of patients would show significant results after systemic anticoagulation alone [15]. This contrasts with another study which suggested a failure rate exceeding 65% with heparin anticoagulation alone [16]. Therefore, for an asymptomatic patient with occlusive, partial to complete thrombosis bowel, a more rapid recanalization is needed by adopting a combined therapy of systemic anticoagulation and CDT [12, 16]. Partial venous recanalization was evidenced in 75 to 100% of PMVT patients receiving the combined therapy with overall excellent success rates [5, 7]. However, more critically symptomatic cases can form significant clinical challenges necessitating alternative, more invasive interventions to reduce the thrombus burden [16]. Therefore, emergency laparotomy, open thrombectomy, and bowel resection should not be delayed in suspected infarction or subsequent perforation [17]. In our two cases, both patients were hemodynamically stable with the presence of mild abdominal tenderness in case # 2, for whom a limited bowel resection was performed after CDT. Both ultimately received combined therapy with trans-hepatic and trans-splenic approaches, which showed effective and safe results in resolving the thrombosis.

CDT for the treatment of PMVT can be performed either by direct trans-venous approach which can be combined with mechanical thrombectomy, balloon angioplasty, and stenting or by indirect trans-arterial approach. The direct approach gives direct access to the portal system through portal, splenic or jugular veins. The percutaneous trans-hepatic technique, usually performed under ultrasound guidance, is a known option to access PMVT. However, it carries the risk of bleeding, primarily if large catheters or sheaths are used during thrombolysis or thrombectomy with hemodynamic instability rates of up to 60% [7]. The ultrasound-guided percutaneous trans-splenic technique is another direct way to access the portal system. However, it still carries a higher bleeding risk than trans-hepatic approaches [18, 19]. Therefore, the decision for this approach should only be taken if the trans-hepatic option is undesirable. This is why we opted for the trans-splenic approach in case # 2, which showed extended intrahepatic portal vein thrombosis in the CECT, making the trans-hepatic puncture of the portal vein branches challenging to obtain under ultrasound guidance.

The trans-jugular intrahepatic portosystemic shunt (TIPS), although technically more challenging, is recommended in the presence of abnormal deranged coagulation, causing less intraperitoneal bleeding than the trans-hepatic or trans-splenic approaches [20].

The second way to proceed with CDT is by an indirect approach using trans-femoral or trans-radial arterial accesses to insert a multi-side hole perfusion catheter into the proximal superior mesenteric artery (SMA), which allows for thrombolytic agents infusion [20, 21]. Nevertheless, this method has a significant limitation as the total dose of infused tPA can result in harmful effects on the surrounding tissues and structures through its cellular toxic effect, locoregional toxicity. In addition, this method requires prolonged infusion time and therefore increases the bleeding complications. Also, in contrast to the direct methods, only thrombolysis and no other adjunctive mechanical interventions are applicable as there is no direct mechanical contact with the clot, but rather the thrombolytic effect is achieved indirectly by the diffusion thrombolytic agents from the adjacent SMA, allowing the agents to the thrombus in the portomesenteric venous system where it drains and does its effect.

Finally, surgical trans-ileocolic approaches were described, offering access to the portomesenteric vein. However, it is less suitable for mechanical techniques like thrombectomy, balloon angioplasty, or stenting [20].

Both patients had regular follow-up by venogram, and the vital signs were continuously monitored during tPA infusion. The duration of tPA infusion was based on the results obtained by the venogram and CECT. The infusion was interrupted once partial recanalization of the portomesenteric vein was obtained to reduce the bleeding risk.

In conclusion, PMVT is a relatively uncommon complication in patients undergoing LSG. However, early detection and treatment based on high clinical suspicion and awareness of clinical presentation and imaging results are necessary to prevent long-term complications. Among the various approaches for recanalizing the PMVT, including thrombolysis and thrombectomy techniques, CDT offers a safe and effective option to restore portal and mesenteric veins patency in symptomatic PMVT patients.

## Data Availability

Not applicable.

## Abbreviations

PMVT: Portomesenteric vein thrombosis
LSG: Laparoscopic Sleeve Gastrectomy
CDT: Catheter-Directed Thrombolysis
ED: Emergency Department
CECT: Contrast-Enhanced Computed Tomography
SC: Subcutaneous
SMV: Superior Mesenteric Vein
LMWH: Low Molecular Weight Heparin
tPA: Tissue Plasminogen Activator
TIPS: The trans-jugular intrahepatic portosystemic shunt
SMA: Superior Mesenteric Artery
